# Does capitation affect the delivery of oral healthcare and access to services? Evidence from a pilot contact in Northern Ireland

**DOI:** 10.1186/s12913-017-2117-3

**Published:** 2017-03-06

**Authors:** Harry Hill, Stephen Birch, Martin Tickle, Ruth McDonald, Michael Donaldson, Donncha O’Carolan, Paul Brocklehurst

**Affiliations:** 1 School of Dentistry, University of Manchester, Manchester, M13 9PL UK; 2 Manchester Centre for Health Economics, University of Manchester, Manchester, M13 9PL UK; 3 Department of Clinical Epidemiology and Biostatistics, McMaster University, Hamilton, ON L8S 4K1 Canada; 4 Manchester Business School, University of Manchester, Manchester, M13 9PL UK; 5 Health & Social Care Board, Belfast, BT2 8BS Northern Ireland; 6 NWORTH Clinical Trials Unit, Bangor University, Bangor, Gwynedd, LL57 2PZ UK; 7 Centre for Health Economics, Institute of Population Health Faculty of Medical and Human Sciences, University of Manchester, Room 4.311, Jean McFarlane Building, Oxford Road, Manchester, M13 9PL UK

## Abstract

**Background:**

In May 2009, the Northern Ireland government introduced General Dental Services (GDS) contracts based on capitation in dental practices newly set up by a corporate dental provider to promote access to dental care in populations that had previously struggled to secure service provision. Dental service provision forms an important component of general health services for the population, but the implications of health system financing on care delivered and the financial cost of services has received relatively little attention in the research literature. The aim of this study is to evaluate the policy effect capitation payment in recently started corporate practices had on the delivery of primary oral healthcare in Northern Ireland and access to services.

**Methods:**

We analysed the policy initiative in Northern Ireland as a natural experiment to find the impact on healthcare delivery of the newly set up corporate practices that use a prospective capitation system to remunerate primary care dentists. Data was collected from GDS claim forms submitted to the Business Services Organisation (BSO) between April 2011 and October 2014. Health and Social Care Board (HSCB) practices operating within a capitation system were matched to a control group, who were remunerated using a retrospective fee-for-service system.

**Results:**

No evidence of patient selection was found in the HSCB practices set up by a corporate provider and operated under capitation. However, patients were less likely to visit the dentist and received less treatment when they did attend, compared to those belonging to the control group (*P* < 0.05). The extent of preventive activity offered and the patient payment charge revenue did not differ between the two practice groups.

**Conclusion:**

Although remunerating NHS primary care dentists in newly set up corporate practices using a prospective capitation system managed costs within healthcare, there is evidence that this policy may have reduced access to care of registered patients.

**Electronic supplementary material:**

The online version of this article (doi:10.1186/s12913-017-2117-3) contains supplementary material, which is available to authorized users.

## Background

Funding the delivery of oral health services in public funded systems is complex and governments wrestle to align financial incentives for health care professionals with policy goals [1]. Economic theory suggests that the method of remuneration influences provider behaviour and a large body of evidence, principally in the field of medicine, supports this theory [2]. Dental service provision forms an important component of general health services for the population, but the implications of health system financing on care delivered and the financial cost of services has received relatively little attention [3]. For example, a 2013 Cochrane Effective Practice and Organisation of Care systematic review examined the impact of different methods of remuneration on the behaviour of primary care dentists and found that the number of available studies that met the eligibility criteria was limited and the quality of the evidence was poor [4].

### Dental service provision in the United Kingdom

In the United Kingdom (UK) context, dental service provision is provided by both the public and private sectors. The private sector is financed by patient charges and/or private insurance. Public dental services are provided under the UK National Health Service (NHS) financed by the Department of Health (via direct taxation) and patient charge revenue. Children, adolescents and exempt adults receive NHS dental treatment free at the point of delivery. Dentists providing NHS services receive Fee-For-Service (FFS) remuneration related to the number and type of dental treatments they provide to adult patients and in Northern Ireland and Scotland a capitation payment for the number of registered children with the practice and allowances such as Continuing Professional Development allowance, General Dental Practice allowance and reimbursement of non-domestic rates.

Healthcare policy makers in the UK are experimenting with alternative payment schemes. The Department of Health in Northern Ireland is currently piloting a new contract with payment by capitation for all activity as an approach to contain costs, promote prevention rather than treatment of disease and improve the quality of care provided to patients [5]. The Department of Health in England is prototyping a new contract with payment by capitation for all activity after the FFS component of the current dental contract came under widespread criticism for providing incentives to dentists to deliver more treatments rather than reducing service needs through preventive care [6–10]. Other UK initiatives which altered remuneration methods were specific to particular patient groups (e.g. the change in the 1990s to treat children wholly on a capitation basis in England).

### The experiment service development initiative in Northern Ireland

In May 2009, an initiative was set up to promote access to NHS dental care in populations that had previously struggled to secure Health and Social Care Board (HSCB) service provision. This involved introducing new dental practices in Northern Ireland. General Dental Service (GDS) contracts for the new practices were capitation based. Capitation remuneration systems reward primary care dentists according to the number of patients registered to a practice, and hence dentists are given an incentive to provide care to new patients and consider the cost of treatment [4]. Oasis Dental Care were among a number of practices that responded to a tender to provide HSBC dental care in Northern Ireland under capitation contracts and in April 2010, they were awarded funding from the Department of Health, Social Services and Public Safety (DHSSPS) to provide 38 primary care dentists working in 14 new practices located in regions where patient access to oral healthcare was limited.

Contracts were awarded for a period of 3 years based on a block contract payment of £5.7 m per year to (make reasonable efforts to) register and maintain the dental health of 57,000 patients by 1st August 2012 [11]. The HSCB in Northern Ireland monitored monthly registrations and held regular monitoring meetings with Oasis Dental Care [11]. The Oasis practices did not achieve this target level of registrations so in year 4, the contract was changed so that for each patient they registered (up to a maximum of 57,000) they received £94.74 per year for looking after their dental health.

## Methods

The aim of this study was to examine how patient outcomes differed between the newly set up Oasis practices paid by capitation, compared to a matched set of control practices paid by traditional FFS payments, after controlling for other patient and practice characteristics. The following research questions were addressed:

1. Do Oasis practices serve a different patient population than control practices (patient selection)?
2. Are patients registered to Oasis practices less likely to receive care than patients in control practices (service coverage)?
3. Do patients who receive care in Oasis practices differ from patients receiving care in control practices in the types of services received (type and mix of treatment received)?
4. Is the cost of delivering dental care to the HSCB higher under capitation as a result of differences in patient-charge revenue raised (system financial viability)?

### Data collection and matching approach

The data used in this study were based on claim forms that primary care dentists are required to submit to the HSCB as part of their contractual regulations. Although Oasis practices were not remunerated for each treatment item provided, they were still required to submit the same documentation as primary care dental practices for auditing purposes. There was no recruitment of humans into the study. Consent was not required because all data was at the dental practice level (no individual dentist or patient information was used) and the Business Services Organisation (BSO) anonymised the practice data before sending it to the research team for analysis.

We compared the 14 Oasis practices to a control group of matched primary care dental practices paid by FFS. Control practices were selected based on practice location. Postcode data of all registered practices with the HSCB for Northern Ireland was used to select the five nearest practices to each Oasis practice. Distance (smallest) from an Oasis practice was chosen as the matching approach as an attempt to control for the oral health care needs of populations being served by the practices. This provided a control group of 70 practices. After screening the data, four of the seventy control practices were removed from the sample because of incomplete claims data. Practices with less than 1000 patients registered at the practice in any month were removed from the sample because this could have indicated a new practice not yet operating at a steady state or that a practice was reducing registrations in advance of practice closure. This resulted in removing one Oasis practice and nine practices from the control group leaving a study sample of 13 Oasis practices and 57 control practices. 89% (51 of 57) of control practices were matched to an Oasis practice in the same postcode district.

The data from the claim forms were pooled over 43 months (April 2011 to October 2014). Seventy practices formed an unbalanced panel dataset of 2971 monthly practice claims (556 Oasis and 2415 control). A full description of Oasis and control group practices sample characteristics is found in Additional file 1.

The analysis covers the period from April 2011 until October 2013, in order to ensure a sufficient number of monthly observations for panel regression. The beginning of the study period is nearly 2 years after the Oasis contract was signed (May 2009) to accommodate the different starting dates for the Oasis practices.

### Variable description

The analysis is based on routinely collected BSO activity data on a range of study variables and potential confounders. The outcome measures (or dependent variables) are patient characteristics, incidence of care amongst registered patients, type and mix of treatment received, and patient charge revenue raised. The measures do not capture private dental healthcare delivered during the study period. However, the HSCB regularly monitored all healthcare activity delivered in Oasis practices to ensure that the proportion of private healthcare to HSCB treatments delivered to patients is close to the average level found in all Northern Ireland practices. The differences between Oasis practices and control practices on these variables are analyzed in a regression with control variables.

The study population was divided into sub-groups for analysis of patient selection. The outcome measures (or dependent variables) were the following patient characteristics (1) the proportion of registered patients who are 60 years of age or older (2) the proportion of registered patients who are 18 years of age or under (3) the proportion of registered patients exempt from HSCB patient charges between 18 and 60 years of age or under. The payment charge exemption categories were: expectant or nursing mothers, Income Support, Job Seekers Allowance, Working Family Tax Credits, and certificates for full or partial help with health care costs. These exemption categories were chosen to capture the proportion of the registered population where there may be higher expected oral health need [12].

The service coverage examined was that received by registered patients during a particular month and the pattern and type of services received. The analysis shows if the registered patient population Oasis practices see the dentist less frequently, have fewer treatment courses or receive less costly treatment than patients in control practices. The outcome measures used to indicate service coverage are the number of unique patients seen (i.e. repeated visits from the same patient is not counted) per 100 patient registrations, the number of treatment courses per 100 registered patients and the monetary value per treatment course. A single treatment course is all the treatment items provided to a patient (including check-up) that is recorded by the primary care dentist on a payment claim form to the HSCB, so each treatment course covers the completion of a treatment plan.

Treatments provided were categorized into different types to compare the case mix profiles of Oasis and control practices. The outcome measures used to indicate type and mix of treatment was the prevalence of particular services (examinations, restorations (fillings), extractions, and scale and polishes) per 100 unique patients seen. As a measure of the prevalence of preventive care we use the number of fluoride varnishes per 100 children registered at the practice. Routine application of fluoride varnish is recommended for children from the age of two in the NHS in England [13].

System financial viability was assessed from patient charge revenue records the primary care dentists submit to the NHS on their payment claim forms. The outcome measures used to indicate system financial viability were the mean patient charge revenue per registered patient and mean patient charge revenue per treatment course. The label financial viability is given to these variables because Oasis practices might generate less charge revenue even after controlling for quantity of work performed. This is because Oasis might deliver a different mix of services to a different mix of patients. As the patient charge revenue differs by type of service (preventive v treatment and treatment type) delivered and type of patient (exempt v non exempt), the mean charge revenue per service delivered may differ between Oasis and matched practices. On our financial sustainability measures, reductions in patient charge revenue associated with the frequency and content of services delivered by Oasis practices under capitation would increase the net cost of dental care to the HSCB, so is an important consideration for commissioners of dental healthcare considering a change in the method of remunerating dentists.

The explanatory variables used in the analysis to control for patient need were the proportion of adult registered patients to registered children, the proportion of exempt patients and the index of multiple deprivation (IMD) score for the practice location. The capacity of the practice to meet needs was measured by the number of dentists working at the practice.

### Analysis

To address the research questions the mean difference between Oasis and control group practices was estimated after controlling for other explanatory variables using an Ordinary Least Squares (OLS) regression model with robust standard errors. OLS is applied to panel data in long panel form (data on *n* practices, over *t* time monthly periods, for a total of *n* × *t* observations), with a number of monthly observations for each practice. These repeated observations of an individual practice cause clustering and standard errors to fall as the number of repeated monthly observations increases. These standard errors at practice level were adjusted (robust standard errors) to control for this in a pooled OLS regression. The estimates from the OLS models are presented as associations as there is the possibility that unobservable influences that mediate the relationship between the explanatory variables and the outcome measures invalidate any conclusions of causality. Panel models were considered as a way of accounting for unobservable influences, but rejected after coefficient testing. Details of the panel estimation approaches undertaken, tests used to compare the modeling approaches and results with different panel estimators are provided in Additional file 1. The specification of the OLS is defined as

$$ {y}_i={\beta}_0+{\beta}_1{\mathrm{I}}_i+{\beta}_2{\mathrm{x}}_i+{\upepsilon}_i $$

For practice i, *y*_*i*_ is an outcome measure, I is a intervention/policy treatment variable measuring whether it is part of the Oasis group or control group, x_*i*_ are a set of practice and patient covariates and ϵ_*i*_ is an error term. The statistical significance and direction of the estimated intervention coefficient indicates if there is a difference between Oasis practices and the control group after controlling for differences in x_*i*_, practice and patient characteristics that were (a) related to the outcome measure but (b) unrelated to whether a practice operates on the Oasis contract. In the patient selection model, the explanatory variables measuring patient-mix (age 60 and above, children and patient need) were not included because they were not independent of the dependent variable (the proportions of registered patients in particular age groups and in particular exemption categories).

The Ramsey Regression Equation Specification Error Test (RESET) was used to test for model mis-specification i.e. whether there was a statistically significant improvement in fit with non-linear combinations of explanatory variables.

Data analysis used Stata software version 11.1. A *p* < 0.05 was used as the threshold for statistical significance and highlighted in the results tables.

## Results

### Descriptive statistics

Table 1 describes practice characteristics and patient selection variables for Oasis and control practices. Oasis practices had greater staffing capacity to deliver service provision compared to the control practices, with an average of 0.89 more dentists active per practice per month. The mean number of monthly treatment items delivered was larger among control practices (922 treatments items) than among Oasis practices (811 treatment items). The mean monthly number of treatment courses delivered to patients was larger for an Oasis practice (463 treatment courses) than a control practice (392 treatment courses) while the mean monetary value of the treatment plans delivered was lower in Oasis practices than in control practices by £10.83. These differences between the two groups were statistically significant at a 5% level.

**Table 1:** Sample differences between Oasis practices and control practices

Variable	Mean values for Oasis practices	Mean values for control practices	Difference in mean values	*P* value of diff.
Practice characteristics				
Number of dentists	4.11	3.21	0.89	*P<0.01*
Regional deprivation (IMD)	19.8	21.2	−1.41	*P<0.01*
Monthly number of treatment items	811	922	−110	*P<0.01*
Monthly number of treatment courses	463	392	71	*P<0.01*
Monthly number of registrations	3850	3359	490	*P<0.01*
Monthly number of patients seen	320	306	14.4	0.15
Case-mix				
% of registered patients age 60 and above	13.3	15.3	−2.01	*P<0.01*
% of child registered patients	19.9	30.7	−10.7	*P<0.01*
% of registered patients exempt from payment charges	36.7	43.2	−6.6	*P<0.01*

All italicized *P* values are statistically significant at a 1% level

### Results from the economic model

The estimated coefficients are presented in Table 2. Each row in the table is a separate regression; with a different dependent variable related to a particular research question. Estimated coefficients on the intervention variables indicate the difference in the outcome variable between Oasis practices and control practices when the values of other explanatory variables in the equations are held constant. The table shows the intervention variable estimate without the use of control variables, and with practice and case-mix control variables. The latter is the main model specification. *P* values of the intervention variable, r^2^, the number of practices (n), the number of practice month observations (N) are presented for each regression.

**Table 2:** Series of regression models to show findings for each research question

Dependent variable	Intervention variable estimate		Estimate with control variables		n, N, r^2^
	Coeff.	*p*-value	Coeff.	*p*-value	
Patient selection					
% of registered patients age 60 and above	−2.01	0.10	-	-	70, 2557, 0.04
% of registered patient that are children	−10.74	*P<0.01*	-	-	70, 2557, 0.19
% of registered patients exempt from payment charges for reasons associated with high dental care need	−6.55	*0.05*	-	-	70, 2557, 0.14
Receipt of care among registrants					
Patients seen per 100 registrations	−0.54	0.07	−1.61	*P<0.01*	70, 2557, 0.11
Treatment courses per 100 registrations	0.78	*0.01*	0.44	0.33	70, 2557, 0.01
Value of treatment per treatment course	−10.83	*P<0.01*	−14.00	*P<0.01*	70, 2557, 0.29
Mix of treatments					
Examination per 100 patients seen	−4.30	*0.02*	−9.7	*P<0.01*	70, 2557, 0.05
Extractions per 100 patients seen	4.62	*P<0.01*	6.31	*P<0.01*	70, 2557, 0.18
Fillings per 100 patients seen	−15.97	*P<0.01*	−17.12	*P<0.01*	70, 2557, 0.22
Scale and polish per 100 patients seen	−8.13	*0.01*	−11.5	*P<0.01*	70, 2557, 0.07
Fluoride varnish per 100 patients seen	−0.01	0.80	0.03	0.41	70, 2557, 0.19
Fluoride varnish per 100 child registrations	−0.001	0.91	0.008	0.33	70, 2557, 0.06
Financial viability					
Patient payment charge revenue per registration	3.93	*P<0.01*	−0.68	0.63	70, 2557, 77
Patient payment charge revenue per treatment course	1.37	0.17	−2.91	*P<0.01*	70, 2989, 0.80

The findings in Table 2 were robust to model specification. The RESET test was performed for each outcome measure. In each case the *p* value of the test was greater than 0.05 indicating that the null hypothesis (that the model is not mis-specified) should not rejected. In addition, there is little difference in the magnitude and statistical significance of the intervention variable when it is estimated by random effects panel model or panel model a Mundlak correction (see Additional file 1).

### Patient selection

There is no evidence of differences in patient mix between Oasis and control practices based on patient need. The proportion of registered patients from payment charge exempt groups with higher expected healthcare need was not significantly different between the two practice types. There was mixed evidence of patient selection based on age. Oasis practices had on average 11 fewer children per 100 registrations than control practices (*P* < 0.00), although there was no statistically significant difference in the number of older (age 60+) registrants per 100 practice registrants.

### Receipt of care among registrants

An Oasis practice saw on average 1.6 fewer unique patients per 100 patient registrations per month than a control group practice (*P* < 0.00). The average value per treatment course was £14.00 lower in an Oasis practice than in a control practice (*P* < 0.00). This suggests that within the given time period, Oasis practices saw a smaller proportion of their registered patients and provided courses of a lower monetary value compared to control practices. There was no statistically significant difference between the practice groups in the average number of monthly treatment courses provided per registered patient, which suggests that there was no evidence of Oasis practices ‘underserving’ registered patients in the absence of any service volume remuneration incentives.

### Mix of treatments

There was evidence of a difference in treatment prescribing patterns between the two practice groups. An Oasis practice provided an average of 9.7 fewer examinations, 17.2 fewer fillings and 11.5 fewer scale and polish services per 100 unique patients seen per month than control practices (*p* < 0.00). However, they provided an average of 6.3 more extractions per 100 unique patients per month (*p* < 0.00). The volume of fluoride varnish applications per patient seen and per 100 child registrations was not significantly different between practice groups.

### Financial viability

In terms of the financial implications of funding care under capitation (the Oasis contract), Oasis practices received an average of £2.91 less patient charge revenue per treatment course than control practices (*P* < 0.00) because of the higher proportion of adult patients exempt from charges. However, this was mitigated by the lower proportion of children in Oasis practices, meaning a greater proportion of the treatment plans delivered by Oasis practices were subject to patient charge payments. Once we adjust for these ‘offsetting’ patient-mix factors, it resulted in no significant difference in the mean monthly payment charge revenue per registered patient between Oasis and control practices.

## Discussion

There was no difference in patient payment charge revenue and the extent of preventive activity offered between Oasis practices under a capitation system and nearby practices on FFS. This is in contrast to findings from other studies, where financial incentives in the remuneration system impacted the amount of clinical activity provided to patients in the NHS [4, 9, 14]. There is evidence of large and abrupt changes in the provision of many types of treatments (Bridge work, crowns, extractions, fillings, root fillings, radiographs) with the change to a new incentive structure in England and Wales in 2006 [4]. Another UK study found that after adjustment for baseline differences, sealant treatment was 9.8% higher when remuneration was by a fee [6]. A major consideration when evaluating the policy of expanding dental healthcare by funding a corporate provider to set up new practices and remunerate those practices by capitation is whether they attract and/or serve populations with different needs to the control practices. Our analysis found no evidence of patient selection (‘or cherry picking’) associated with different patient age groups with broadly differing oral health care needs among Oasis practices.

Oasis practices were purposely set up in locations where the government had identified barriers which prevented access to oral healthcare in the resident population. The policy aim of the pilots was to improve access in these areas and Oasis practices largely met the target number of 57,000 registered patients set by the government. However, registered patients were also found to be less likely to visit a primary care dentist compared to patients in neighbouring control group practices (1.6 fewer unique patients per 100 patient registrations per month) and received less treatment when they did attend (the average value per treatment course was £14.00 lower). These are similar to findings in other studies that have investigated dental practice remuneration methods. A US study found an average of 9.41 treatment items for dentists under FFS patients compared to 3.69 for those under capitation [15], another study found the average cost of a treatment course under capitation was $131.76 compared to $169.90 with FFS [16] and the mean number of visits per 0 to 15 year olds has been found to be lower in regional areas where dentists were under capitation contract compared to FFS contract areas [17]. Our findings suggests either less over-provision under capitation in Oasis practices or more under-provision, although it is important to note that over 300 Oasis patients were examined by independent dentists to check care quality and to look for under/over provision. They found the care was comparable to the GDS and there was no systematic under or over provision. Instead the findings of fewer visits and less treatment in a treatment course might be the result of unobserved heterogeneity between practices. For example, Oasis practices were set up where populations were unable to get access to a NHS dentist and so it may be that some individuals in the Oasis practice population had their care needs met by the private sector. If this were the case, differences in service coverage would not be caused by the different ways the dentists were remunerated or different practice structures (the characteristics of the dentists, management and organization of practice staff).

Ideally, an evaluation of the pilot contract would incorporate considerations of the quality of products and services in terms of impact on patient outcomes (e.g. oral health and satisfaction with care). The absence of these measures means that our study findings should be interpreted with caution. The lower number of unique patients seen by Oasis practices (per 100 patient registrations) may be a reflection of higher-quality care that produces better patient outcomes by, for example, taking more time with each patient seen. However it may also reflect lower patient satisfaction with the quality of care delivered, causing fewer patients to visit the practice. Further exploration of this line of enquiry would require data on quality of care.

We try to control for major components of geographical heterogeneity between Oasis and FFS practices. The model included explanatory variables that captured the age mix of the practice population, the prevalence of exemption from dental payment charges for reasons of higher risks of oral health problems and the level of deprivation in the local community. The later variable is of limited use for capturing the socioeconomic deprivation of patients because patients can choose to attend a practice from any geographic region. We try to overcome this problem by using the outward code of practice postcode districts for the regional deprivation indicator. The outward code covers regions the size of a town so it may be that few patients travel to a practice in another postcode region (and hence deprivation area) particularly because Oasis practices were located in areas with fewer providers (the purpose of the policy was to increase availability of care in areas of poor dental service access). Our analyses of practice level data could still be biased if the populations of practices differ significantly in other relevant factors than the ones controlled for. Hence, we chose a method of matching to reduce the likelihood that differences in the practice level measures have arisen by the nature of patients by having patients drawn from the same locality. These methods reduce but may not remove all between-practice variations in patient populations.

Causality is not established by the modelling approach although the service coverage findings were consistent with dentists working under capitation; seeing patients less frequently and ‘doing less’ to patients when they do see them. Similar findings were reported in other studies. A US study found that capitation practices provided less-expensive services [15], an average of 3.62 crown and bridge services were planned for adult FFS patients compared to 0.42 by capitation, and studies from the UK, Sweden, Denmark and Norway found that dentists with per capita remuneration ‘under-treated’ patients [17–20].

Capitation introduces an incentive for clinicians to choose treatment options that require less time [21]. In terms of the type and mix of services delivered, the higher prevalence of extractions in Oasis practice (6.3 more extractions per 100 unique patients per month) could be because extractions take less time than treatment alternatives (for example, root canal treatments). The lower prevalence of examinations and fillings among Oasis practices (9.7 fewer examinations and 17.2 fewer fillings per 100 unique patients seen per month) suggests that patients registered with these practices were not seen as often and had longer recall periods. Although this might reduce the prevalence of unnecessary interventions among patients, equally it may also lead to some oral health problems being missed and interventions being delayed. These findings are in contrast to those found in Sweden where capitation was found to increase the number of examinations [22] and in Norway, where the finding of no under-diagnosis of carious lesions under capitation suggests health problems were not missed [19]. However, the majority of studies find results similar to that in the Oasis group with the number of restorative services being less under capitation [23–25].

Capitation introduces an incentive for clinicians to meet health need through preventive care rather than more treatment [9]. We found no evidence of this in the Oasis practices, which were as likely to use preventive treatments on patient population than the control group. This finding should be interpreted with caution because preventive care was measured with treatments that could have been desirable to patients for reasons other than extent of prevention offered. For example, “scaling and polishing” aims at removing calculus and plaque that can cause oral health problems (periodontal disease) in the future although it is a procedure that many patients like, due to its aesthetic value (removal of stained deposits). For this reason, the application of fluoride varnish is potentially a better marker of prevention orientation. However, its provision may be influenced by the absence of FFS for fluoride varnish whereas there is a fee for fissure sealants (the fluoride varnish application is covered by the capitation payment as the patient group is children). The few studies that investigate the effect of capitation payment in dentistry on service-mix in the UK focus on a pilot contract that lasted from 1984 to 1989 and a capitation scheme for child patients from 1990 [23, 25–29]. They find more preventive procedures carried out by dentists with per capita remuneration than by dentists with FFS. One of these studies specifically aimed to investigate the levels of restorative care in three England regions where dentists under FFS remuneration changed to capitation, they found 5 years later that the prevalence of preventive care treatment (fissure sealants) increase from 16 to 30%, 13 to 50% and 25 to 47% respectively [29]. This in in contrast to what was found in Oasis practices although the difference in results may be explained by Oasis practices receiving capitation remuneration for adult and child patients while the previous studies examined a capitation scheme for children only. In addition, the difference in results may be because the Oasis practices were recently started by a corporate provider while the practices examined in previous studies are likely to have been under ownership from a individual or partner group of dentists and have been operational for a range of periods before the evaluation of their performance took place. Although Oasis practices were recently started, the study period started around 2 years after the Oasis contract was signed. In this time newly registered patients received treatment. Hence, by the time the study commenced these patients’ will have had 2 years exposure to services with accumulated needs for treatment having already been addressed, at least in part, prior to the period of data analysis.

Finally, there is evidence that remuneration by capitation in recently started corporate practices is as financially viable to NHS commissioners of healthcare as current services. This is because there was no difference in practice revenues transferred to HSCB commissioners (accrued in practices from treatment fees charged to patients) per unit of service coverage (registrations) and per unit of healthcare delivered (treatment plans). The patient payment charge revenue per registered patient did not differ between Oasis practices and those that operated on a FFS basis. However, Oasis practices had a smaller percentage of children (who are exempt from NHS patient charges) compared to control practices by a mean difference of 10.7%, which would offset the reduced revenue per patient income and may explain the non-significant finding in overall patient payment charge income. In the literature, only one study specifically aimed to investigate the financial impactions of a capitation scheme and it found a net positive economic outcome over 3 years for a pilot contract in a Swedish dental clinic [22].

The main limitation of the study concerned the limited amount of information available on the practices and the populations served by the practices, as well as the non-random nature of allocating the provider payment methods and allocation of patients. Receipt of oral healthcare is conditional on registration therefore the models used in this study were conditional on registrations and hence subject to selection bias. One approach to correct for sample selection is the Heckman model [30]. This incorporates the probability of registration within the population, but as this study is confined to *practice* populations, such methods cannot be used. Instead an OLS regression model with robust standard errors was used. As highlighted above, the study was designed to minimise the impact of patient and practice heterogeneity between Oasis and control practices. As a result, a ‘difference in difference’ design could not be used (there was no ‘before intervention’ period for the Oasis practices) [31]. Although we were able to identify statistically significant differences in some aspects of patient populations, receipt of care amongst registrants, service mix and patient payment charge revenue, data was not available on patient outcomes. As such, it is not possible to determine whether the lower prevalence of interventions in the Oasis practices, was the result of inappropriate supplier induced demand among control practices (unnecessary interventions) or the failure to intervene in a timely way among Oasis practices (supervised neglect) or both.

In addition to the selection bias of patients, another study limitation was the possible selection bias in the allocation of practices to receive the capitation contract. This was non-random, and the Oasis practices could be expected to operate under a different management system. This is because the intervention practices were owned by a single private limited company responsible for over 300 dental practices in the UK while the control group practices were owned by single dentist or a small team of owner dentists and/or partners. This makes it difficult to separate the effect of financial incentives from the effect of ownership model and management culture. The practices were in areas where access to HSCB dentistry was problematic, which was the reason why the Department introduced the capitation scheme. Hence, the findings may not be generalisable to areas already well served with practices providing HSCB dental care. Another limitation is that it was not possible to discern whether our findings are caused by differences between Oasis practices and controls in practice structures (characteristics of dentists, method of remuneration). In addition, aggregate differences between Oasis practices and controls may have been caused by changes in the behavior of dentists in Oasis practices in response to their awareness of being monitored by the DHSSPS (the ‘Hawthorn effect’). This is because the DHSSPS were required to check if Oasis practices had met a contracted minimum number of patient registrations, dentist working hours and proportion of HSCB healthcare to private sector care delivered to patients.

## Conclusion

This study explored the impact of policy that set up practices with different systems of managing NHS dentist’s remuneration in Northern Ireland by analyzing the policy intervention as a natural experiment. There is evidence the payment charge revenues the NHS received from capitation in recently started corporate provider practices was not different from the payment charge revenues the NHS received from FFS providers, suggesting the policy was financially viable to the commissioners of dental healthcare. There is some evidence of change to the organisation of the healthcare output in dental practices. The pilot practices examined fewer patients and undertook fewer restorations. The pilot practices under capitation remuneration did not have higher levels of prevention, but did for the proportion extractions undertaken. These results must be interpreted with caution as the implications of these differences for patient outcomes are not clear. Rather than using observational data, a definitive randomised controlled trial would be required to understand whether differences in the remuneration system was causally related to patient outcomes.

## Additional file

Additional file 1: The document does not contain data. It contains descriptive statistics of the Oasis and control group, detailed explanation for the chosen panel estimation approach and results with different panel estimators. (DOCX 29 kb)

## Abbreviations

BSO: Business Services Organisation
DHSSPS: The Department of Health, Social Services and Public Safety
FFS: Fee-For-Service
GDS: General Dental Services
HSCB: Health and Social Care Board
IMD: Index of multiple deprivation
OLS: Ordinary Least Squares
RESET: The Ramsey Regression Equation Specification Error Test
UK: United Kingdom
