# Supplementary material for: Does capitation affect the delivery of oral healthcare and access to services? Evidence from a pilot contact in Northern Ireland
Source: BMC Health Serv Res. 2017 Mar 6;17:175. doi: 10.1186/s12913-017-2117-3 (PMC5339966; doi:10.1186/s12913-017-2117-3)
Supplement: Additional file 1: — The document does not contain data. It contains descriptive statistics of the Oasis and control group, detailed explanation for the chosen panel estimation approach and results with different panel estimators. (DOCX 29 kb) [file 12913_2017_2117_MOESM1_ESM.docx]

**Additional file 1**

**This section of the additional file contains the descriptive statistics of the Oasis and control group.**

Table 1: Sample characteristics for receipt of care, treatment-mix and financial viability variables

| **Variable name** | **Average for a control practice** | | **Average for an Oasis practice** | | **Difference** | |
| --- | --- | --- | --- | --- | --- | --- |
|  | **Mean (n*)** | **95% CI** | **Mean (n*)** | **95% CI** | **Mean diff.** | **p- value** |
| **Receipt of care among registrants** |  |  |  |  |  |  |
| Monthly number of patients seen per 100 registrations | 8.9 (2415) | 8.8 - 9 | 8.4 (556) | 8.1 - 8.6 | 0.54 | **<0.00** |
| Monthly number of courses per 100 registrations | 11.7 (2079) | 11.6 - 11.9 | 12.5 (478) | 12.1 - 12.9 | - 0.8 | **<0.00** |
| Value of treatment per treatment course | 33.8  (2079) | 33.4 - 34.2 | 22.9 (478) | 22.4 - 23.5 | 10.8 | **<0.00** |
| **Mix of treatments** |  |  |  |  |  |  |
| Monthly number of examinations per 100 patients seen | 66.7 (2079) | 66.2 -  67.1 | 62.4 (478) | 61.6 - 63.2 | 4.30 | **<0.00** |
| Monthly number of extractions per 100 patients seen | 16.1 (2079) | 15.8 - 16.4 | 20.7 (478) | 20 - 21.4 | - 4.6 | **<0.00** |
| Monthly number of fillings per 100 patients seen | 60.3 (2079) | 59.6 - 60.9 | 44.3  (478) | 43.3 - 45.4 | 15.9 | **<0.00** |
| Monthly number of Scale and polishes per 100 patients seen | 48.2 (2079) | 47.6 - 48.7 | 40 (478) | 39 - 41.1 | 8.1 | **<0.00** |
| Monthly number of Fluoride varnishes per 100 patients seen | 0.023 (2079) | 0.015 - 0.032 | 0.018 (478) | 0.008 - 0.029 | 0.005 | 0.60 |
| Monthly number of Fluoride varnishes per 100 child registrations | 0.008  (2079) | 0.005 - 0.01 | 0.007 (478) | 0.003 - 0.01 | 0.0007 | 0.83 |
| **Financial viability** |  |  |  |  |  |  |
| Patient payment charge revenue per registration | 11.5 (2079) | 11.1 - 11.8 | 15.4 (478) | 15.1 - 15.7 | - 3.93 | **<0.00** |
| Patient payment charge revenue treatment course | 9.1 (2079) | 8.8 -  9.41 | 10.5 (478) | 10.2 - 10.8 | - 1.4 | **<0.00** |

* one observation is one practice observed in one month

The revenues accrued to the practice from patient payment charges per registration and per patient seen are both higher for an Oasis practice by £3.93 and £1.37 respectively. Oasis practices delver an average of 4.6 more extractions per 100 patients seen than control practices. Other types of treatments are less frequently delivered in Oasis practices. These differences are statistically significant which indicates different service mixes between Oasis and control practices.

**This section of additional file 1 details the panel estimation approaches undertaken and results with different panel estimators.**

Panel models provide a way of accounting for unobservable influences, but the data available for this analysis did not favor that approach because of a lack of variation over time. Panel model specifications were estimated, tested with an F-test and a Lagrange multiplier test and rejected in favor of pooled OLS. The coefficients and statistical significance of exploratory variables in the OLS model were almost identical to panel specifications and a joint Wald-test did not reject the null hypothesis that the coefficients on time-varying explanatory variables were identical. This suggests there was not enough variation across the months within practices to justify a panel approach and we chose pooled OLS with robust standard errors as our main model specification.

A limitation of the pooled OLS approach is the potential bias for unobserved individual heterogeneity on estimators. Each dental practice has its own unique individual characteristics not all of which are observed in the data but which may influence or bias the explanatory variables. For example, the tenure of dentists could influence the amount of healthcare they provide, the number of nearby dental practices could influence the amount of patient registrations, business expertise may influence the size of the practice; use of DCPs could influence the treatment service-mix. Not controlling for unobserved practice specific effects leads to potential bias in the resulting estimates. In panel estimation, the influence of time-invariant practice characteristics is assumed to be captured by the correlation between the error term for the practice and explanatory variables.

Fixed and random effects (RE) estimators are alternative estimators for the panel model. Each has advantages and disadvantages. However in this study random effects is appropriate because calculating an estimate of the intervention variable (the estimated coefficient of intervention variable indicates the effect on an outcome of belonging to either the Oasis or control group) is not possible with a fixed effects estimator as that variable is time-invariant. We use the Hausman test to test the null hypothesis that the coefficients estimated by a more efficient model (Random effects) are the same as the ones estimated by a less efficient but consistent model (Fixed effects). At a 5% significance level the null hypothesis was accepted which means that random effects are preferable to fixed effects.

A problem with the random effects model is that it assumes that the practice specific effect is a random variable that is uncorrelated with the explanatory variables of all past, current and future time periods of the same practice. This assumption is false if for example the ability of a practice to deliver oral healthcare is learnt from their experience delivering care to patients. Then the practice specific effect would not be random but related to types of patients they have seen (patient case mix explanatory variables). The assumption would also be false if the ability of a practice to deliver healthcare arises from its management structure, organisational culture and workplace environment. Those are in part constituted by the number and type of staff the practice has employed so the practice specific effect would be correlated with the explanatory variables measuring staffing levels.

To relax this assumption we estimate the RE model with a Mundalk correction. This approach is often used with random effects models and is conducted by adding group means of the time-varying explanatory variables to the model. The results of the estimators are now divided between the current effect and long term effect of the explanatory variables on the outcome variable. The coefficient on the group means of the time-varying explanatory variables is the longer term effect of those variables on the current outcome, while the coefficient on the time-varying explanatory variables is the current effect of those variables on current outcome. The current effect of the time-varying explanatory variables should be less than the ‘total’ (current and long term) effect if the model holds true. Hence, if the correct specification is the Mundlak corrected RE model the coefficients on its time-varying explanatory variables (current effect) should be of smaller magnitude than the coefficients in the OLS model and RE model on the time-varying explanatory variables (‘total’ effect). Table 2 shows the coefficients and statistical significance of exploratory variables to be almost identical across the three model specifications. A joint Wald-test did not reject the null hypothesis that the coefficients on time-varying explanatory variables are identical. This suggests there is not enough variation across the months within practices to justify a panel approach and we chose pooled OLS with robust standard errors as our main model specification.

Table 2: Results using OLS with robust standard errors, RE and RE with the Mundlak correction

| **Dependent variable (i.e. outcome measure)** | **OLS** | | **RE** | | **RE with Mundlak** | |
| --- | --- | --- | --- | --- | --- | --- |
|  | **Intervention variable estimate** | **p-value** | **Intervention variable estimate** | **p-value** | **Intervention variable estimate** | **p-value** |
| **Patient selection** |  |  |  |  |  |  |
| % of registered patients age 60 and above | - 1.72 | P=0.35 | - 2.28 | P=0.20 | - 1.73 | P=0.35 |
| % of registered patient that are children | - 11.2 | **P=<0.00** | - 11.04 | **P<0.00** | - 11.16 | **P<0.00** |
| % of registered patients exempt from payment chrages for reasons associated with high dental care need | - 3.81 | P=0.21 | - 3.78 | P=0.50 | - 3.56 | P=0.53 |
| **Receipt of care among registrants** |  |  |  |  |  |  |
| Patients seen per 100 registrations | - 1.61 | **P<0.00** | - 1.85 | **P<0.00** | - 1.51 | **P<0.00** |
| Treatment courses per 100 registrations | 0.69 | P=0.25 | - 0.22 | P=0.70 | 0.69 | P=0.25 |
| Value of treatment per claim | - 14.29 | **P<0.00** | - 14.44 | **P<0.00** | - 14.29 | **P<0.00** |
| Mix of treatments |  |  |  |  |  |  |
| Examination per 100 patients seen | - 9.76 | **P<0.00** | - 9.50 | **P<0.00** | - 9.76 | **P<0.00** |
| Extractions per 100 patients seen | 6.20 | **P<0.00** | 7.18 | **P<0.00** | 6.20 | **P<0.00** |
| Fillings per 100 patients seen | - 17.89 | **P<0.00** | - 16.90 | **P<0.00** | - 17.89 | **P<0.00** |
| Scale and polish per 100 patients seen | - 11.47 | **P<0.00** | - 14.45 | **P<0.00** | - 11.47 | **P<0.00** |
| Fluoride varnish per 100 patients seen | 0.034 | P=0.41 | 0.017 | P=0.66 | 0.034 | P=0.41 |
| Fluoride varnish per 100 child registrations | 0.008 | P=0.53 | 0.005 | P=0.65 | 0.008 | P=0.53 |
| **Financial viability** |  |  |  |  |  |  |
| Patient payment charge revenue per registration | - 0.53 | P=0.63 | - 1.42 | P=0.14 | - 0.53 | P=0.63 |
| Patient payment charge revenue per treatment course | - 2.85 | **P<0.00** | - 3.02 | **P<0.00** | - 2.85 | **P<0.00** |
